# Protective Effect of Virgin Coconut Oil on Osteopenia Induced by High Refined Carbohydrate-Containing Diet in Mice

**DOI:** 10.3390/foods11182800

**Published:** 2022-09-11

**Authors:** Marina C. Zicker, Carina C. Montalvany-Antonucci, Débora R. Lacerda, Marina C. Oliveira, Tarcília A. Silva, Soraia Macari, Mila F. M. Madeira, Adaliene V. M. Ferreira

**Affiliations:** 1 Immunometabolism, Department of Nutrition, Nursing School, Universidade Federal de Minas Gerais, Belo Horizonte 30130-100, Brazil; 2 Department of Oral Surgery and Pathology, Faculty of Dentistry, Universidade Federal de Minas Gerais, Belo Horizonte 31270-901, Brazil; 3 Department of Restorative Dentistry, Faculty of Dentistry, Universidade Federal de Minas Gerais, Belo Horizonte 31270-901, Brazil; 4 Department of Microbiology, Instituto de Ciências Biológicas, Universidade Federal de Minas Gerais, Belo Horizonte 31270-901, Brazil

**Keywords:** virgin coconut oil, high refined carbohydrate diet, obesity, bone loss, alveolar bone, metabolism

## Abstract

Background: Obesity leads to chronic low-grade inflammation, promoting detrimental effects on bone. The consumption of virgin coconut oil (VCO) is associated with benefits related to meta-inflammation. We evaluated the effect of VCO supplementation on osteopenia promoted by diet-induced obesity in mice. Methods: Male BALB/c mice were fed a control (C) or highly refined carbohydrate-containing (HC) diet for eight weeks. After that, the HC diet group was supplemented with three doses of VCO for four weeks. Results: The HC diet increased the adiposity and leptin levels associated with augmented systemic inflammatory cells improved with VCO supplementation. The HC diet reduced the trabecular bone in the tibia, lumbar vertebrae, distal and proximal femur, as well as the bone mineral density of the femur and alveolar bone. The VCO supplementation reverted bone osteopenia by increasing the trabecular bone in different sites and improving femur and alveolar bone microarchitecture. Although the reduced number of osteoblasts in the alveolar bone of the HC diet group was not significantly enhanced by VCO supplementation, the reduced Alp expression in the HC diet group was enhanced in the VCO group. These beneficial effects were associated with lowering the Rankl/Opg ratio. Conclusion: VCO supplementation might be an effective strategy to attenuate bone osteopenic effects induced by obesity.

## 1. Introduction

Obesity is a systemic disease characterized by body fat accumulation that drives chronic low-grade inflammation with increased production of proinflammatory cytokines and reactive oxygen species. It also leads to other metabolic dysfunctions, including insulin resistance, dyslipidemia, cardiovascular disease, and bone remodeling disorders [1,2,3]. Previous experimental and clinical studies have shown that obesity interferes with bone organic matrix mineralization, leading to bone fragility and, consequently, a higher risk of bone fractures [4,5,6]. Bone detrimental effects caused by obesity are observed not only in long bones (i.e., femur and tibia) [4,5,6,7,8,9] but also in the alveolar bone [10,11,12,13,14,15,16,17]. Obesity-increased alveolar bone loss is known to have an essential role in the development of periodontitis [11,14,15,16,17], an infectious disease characterized by inflammation and destruction of tooth-supporting structures [16].

Current knowledge demonstrates that bioactive dietary substances promote the improvement of oxidative stress and inflammation seen in several undesirable health conditions, including obesity [18], osteoporosis [19,20], and alveolar bone loss [21,22,23]. Hence, dietary strategies may help to promote beneficial effects and attenuate bone dysfunctions induced by obesity. Virgin coconut oil (VCO) has become a food of interest as a natural therapeutic product due to its antioxidant compounds [24,25,26,27,28] and medium-chain fatty acids (fatty acids with 6–12 carbons) content [24]. VCO is obtained from fresh, mature coconut kernel without heating or refining processes [24], which avoids fat degradation and promotes higher retention of phenolic compounds and antioxidant vitamins [24,25,26,28,29]. VCO supplementation has been shown to develop a series of body health benefits, such as improvement of serum lipids [30,31,32,33], glycemic homeostasis [34], and antioxidant status [27,28,30]. These effects seem to be due to its anti-inflammatory properties [35] and body fat reduction [27,32]. Previous studies demonstrated that VCO supplementation improved inflammation in ligature-induced periodontal disease [36], maintained long bone microarchitecture in a model of estrogen-deficient rats [37], and improved the antioxidant defense system in the tibia [38]. Altogether, VCO intake could be correlated with beneficial bone effects and long bone remodeling. However, to the best of our knowledge, studies evaluating the influence of VCO supplementation on alveolar bone metabolism are absent.

In mouse models, the consumption of a high-carbohydrate diet (HC) causes obesity and correlated comorbidities, such as inflammation, metabolic disturbance [39], and detrimental effects on long [40] and maxillary bones [41,42,43]. Previous data showed that VCO supplementation improved obesity features such as a higher adiposity gain as well as metabolic and inflammatory responses [30,32,34,43,44,45,46]. Although a study with rats fed an HF diet and treated with VCO worsened metabolic changes compared with those that received only the HF diet [47], we hypothesized that the amelioration in obesity caused by VCO supplementation promotes benefits in bone health, increasing the perspective of treatments for damaged bone caused by diet-induced obesity in humans. Therefore, the purpose of the present study was to evaluate the impact of VCO supplementation on bone detrimental effects induced by the HC diet in mice.

## 2. Materials and Methods

### 2.1. Mice and Diet 

Male BALB/c mice (6–8 weeks of age—*n* = 40) were obtained from the animal care center of Universidade Federal de Minas Gerais (Bioscience unit-CEBIO-UFMG). The local Ethics Committee in Animal Experimentation approved the experimental protocol (protocol no. 174/2012). Animals were maintained according to the ethical guidelines of our institution and the Guide for the Care and Use of Laboratory Animals. All efforts were made to minimize animal suffering and to reduce the number of animals used. 

Mice were housed under standard conditions (25.4 ± 3.4 °C, with a light-dark cycle of 12 h–12 h) in separated and appropriate cages with free access to commercial chow and tap water. The control diet consisted exclusively of a chow diet (PURINA-LABINA, São Paulo, SP, Brazil) and its content was composed of 65.8% carbohydrate, 3.1% fat, and 31.1% protein with 4.0 kcal/g. The HC diet contained 45% condensed milk, 10% refined sugar, and 45% chow diet composed of 74.2% carbohydrate (at least 30% refined sugars, mostly sucrose), 5.8% fat, and 20% protein with 4.4 kcal/g [39].

An organic VCO was obtained from Finococo^®^ (Conde, Bahia, Brazil), identified, stored under refrigeration (4–10 °C), and protected from light until it was used. 

### 2.2. Experimental Design and Sample Collection

Mice were randomly assigned to two groups for eight weeks fed with either (i) a control diet (C group, *n* = 8), or (ii) the HC diet (*n* = 32). After this period, animals fed with the HC diet were redistributed equally into four groups. One group received the HC diet (HC group, *n* = 8), and the other three groups received the HC diet supplemented with VCO at 1000 mg/Kg (low dose of VCO—LVCO group, *n* = 8), 3000 mg/Kg (medium dose of VCO—MVCO group, *n* = 8) and 9000 mg/Kg of body weight (high dose of VCO—HVCO group, *n* = 8), as previously demonstrated [44]. We verified that the mean body weight was statistically not different for each experimental group before assignment to diet treatment. This time point was chosen to start the supplementation as we previously demonstrated the kinetic of metabolic and inflammatory alterations triggered by HC diet, and at this period, the chronicity appears to have been established [39]. The supplementation was adjusted weekly based on the changes in food intake and body weight for the following four weeks, reaching twelve weeks as an experimental period for all groups [44].

Throughout the experiment, the weight of mice was measured once a week, and food consumption was assessed twice a week. At the end of the dietary treatment, animals were anesthetized with ketamine (130 mg/kg Body Weight) and xylazine (0.3 mg/kg Body Weight) and euthanized with exsanguination. Epididymal (EAT), mesenteric (MAT) and retroperitoneal (RAT) white adipose tissues were collected, weighed and stored at −80 °C for further analysis. The adiposity index was calculated using the formula: [(EAT + RAT + MAT) / body weight in grams] multiplied by 100. Blood was collected to obtain serum which was used for later analysis. The fasting serum levels of leptin were determined by enzyme-linked immunosorbent assay (ELISA, R&D systems Europe Ltd., Abington, UK). The assay was performed according to the procedures supplied by the manufacturer. Samples of bones (maxilla, femur, lumbar vertebrae, and tibia) were obtained and stored in 10% buffered formalin or stored at −80 °C. 

### 2.3. Total and Differential Blood Cell Counts

Blood samples were taken from the animal’s tail, diluted in Türk’s solution, and a Neubauer chamber was used to determine the total leukocyte count. Peripheral blood smears were stained with May–Grünwald–Giemsa (Panoptic kit, Laborclin, Pinhal, Brazil) and the differential white blood cell count was determined under oil immersion (1000X) using standard morphologic criteria.

### 2.4. Histomorphometry of Bone Tissues

Histomorphometry analysis of bone tissues was determined as previously described [42]. Lumbar vertebrae (L1–L6) and long bone (femur and tibia) samples (n = 8 per group) were fixed in 10% buffered formalin, decalcified in 14% ethylenediaminetetraacetic acid (pH 7.4) for 21 days, washed, and embedded in paraffin. The proximal femur was sectioned in the middle diaphysis, and all vertebral segments, femur, and tibia were sectioned longitudinally. Thick sections (4 to 5 µm) were stained with Haematoxylin and Eosin and examined using a light microscope. The percentage of trabecular bone was determined at 40x magnification using an ocular micrometer containing a 121-point grid. Four fields were chosen right below the epiphyseal plate in each bone.

Sections of the maxilla were also stained with Masson’s Trichrome. The osteoblast number per bone perimeter (N.Oc/B.Pm) was determined using ImageJ software (NIH Image, Bethesda, MD, USA). Osteoblast number and the trabecular bone perimeter were quantified in the analysis region. Osteoblast density was calculated by dividing the number of counted cells (#)/the perimeter of the trabecular bone (mm).

### 2.5. Micro-Computed Tomography Analysis 

Micro-computed tomography (Micro-CT) analyses of maxillae and femur were determined as previously described [48]. Maxillae and femur samples (*n* = 5 per group) were fixed in 10% neutral buffered formalin for 48 h and scanned using a micro-CT system (Skyscan 1172 X-ray microtomography; Skyscan, Aartselaar, Belgium). The calibration was carried out with known density calcium hydroxyapatite phantoms (Skyscan). High-resolution images with an isotropic voxel size of 8.62 were acquired (50 kV, 0.5 mm aluminum filter), and the trabecular bone in the furcation area of upper first molars and the metaphyseal region of femurs with a uniformly shaped region of interest were delineated. The tissue was analyzed to determine the bone mineral density (BMD), percent of bone volume/tissue volume (BV/TV), trabecular thickness (Tb.Th), trabecular number (Tb.N), and trabecular bone pattern factor (Tb.Pf). Alveolar bone loss was measured as previously described [49] by determining the area between the cement–enamel junction (CEJ) and the alveolar bone crest (ABC), (CEJ-ACB), in three-dimension images (Fiji—National Institute of Health, Bethesda, MD, USA) of the first, second and third maxillary molars.

### 2.6. Mechanical Analysis

Mechanical analysis of the femur was determined as previously described [50]. Maximum load (Lmax) and stiffness (St) were determined by testing the right femur to fracture in a universal testing machine (EMICs, DL 10000, Ribeirão Preto, Brazil) equipped with a load cell of 500 N, and TESC software version 13.4 (EMIC) (São José dos Pinhais, PR, Brazil). Femurs were tested by the three-point bending flexural test, with force applied at a speed of 1.0 mm/min in the anterior–posterior direction. The gap between the two points was 8 mm, and a 2N preload was used for 30 s. 

### 2.7. mRNA Extraction and qPCR in the Maxilla

For qPCR analysis, the periodontal ligament was removed, and the surrounding alveolar bone was used. Total RNA was extracted using Trizol reagent followed by column purification (RNeasy Mini Kit; Qiagen, Valencia, CA, USA). According to the manufacturer’s instructions, the integrity of RNA samples was checked by analyzing 1 µg total RNA on a 2100 Bioanalyzer (Agilent Technologies, Santa Clara, CA, USA). Complementary DNA (cDNA) was synthesized from 2 µL RNA using the Quanti TectRT kit (Qiagen). The target genes analyzed were: receptor activator of nuclear factor κB Rank (Tfnrsf11a), Rankl (Tnfsf11), Opg (Tnfrsf11b), Runx2 and Alp, using Gapdh as the housekeeping gene.

### 2.8. Statistical Analysis 

Results are expressed as means ± SEM and were analyzed using GraphPad Prism version 5.0 (GraphPad Software, San Diego, CA, USA). All data were analyzed for normality of distribution using the Kolmogorov–Smirnov test and were found to be normal. A comparison between two groups was performed using Student’s *t*-test, and multiple comparisons were performed using one-way ANOVA with a Student–Newman–Keuls posthoc analysis. The calculation of sample size was performed with Gpower Software, version 3.1.9.7, (Franz Faul, Christian-Albrechts-Universität Kiel, Kiel, Germany). To calculate the sample size, we used one-way ANOVA with 5 experimental groups, effect size = 0.75, α error = 0.05, and statistical power = 0.95. The total sample size was 40 animals, with 8 mice in each group. All tests and analyses were performed by investigators blinded to the procedures. Grubbs’ test was performed to determine outliers among the samples, and values statistically lower than 0.05 were considered atypical and excluded from analyses. Statistical significance was set at *p* < 0.05.

## 3. Results

### 3.1. VCO Supplementation Reverses the Obese Phenotype and the Appendicular and Axial Bone Loss Induced by the HC Diet

Even though the body weight gain (data not shown) was similar between the groups, the HC diet consumption increased the adiposity index and leptin serum levels. The consumption of HC diet also promoted a higher number of leukocytes, mononuclear cells and neutrophils in the blood (Table 1). On the other hand, VCO supplementation in all administered doses reverted these effects (Table 1).

**Figure 1:**
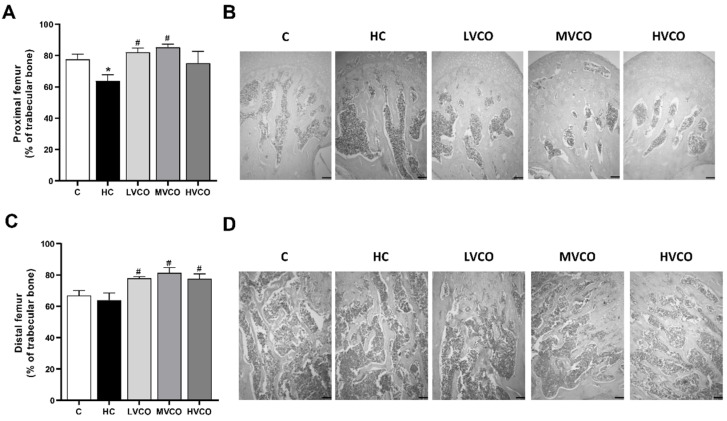
Effect of the HC diet and VCO supplementation on the percentage of trabecular bone in the proximal and distal femur. (**A**) The trabecular bone area and (**B**) histological sections of the proximal femur (scale bars represent 100 µm). (**C**) The trabecular bone area and (**D**) histological sections of the distal femur (scale bars represent 100 µm). Analyses were performed in mice fed a chow diet, high refined carbohydrate-containing (HC) diet, and HC diet supplemented with 1000 mg/kg (LVCO), 3000 mg/kg (MVCO), or 9000 mg/kg (HVCO) of body weight of virgin coconut oil (VCO). Values are means ± SEM (*n* = 8). * compared with the control (C) group (*p* < 0.05); # compared with HC group (*p* < 0.05).

**Figure 2:**
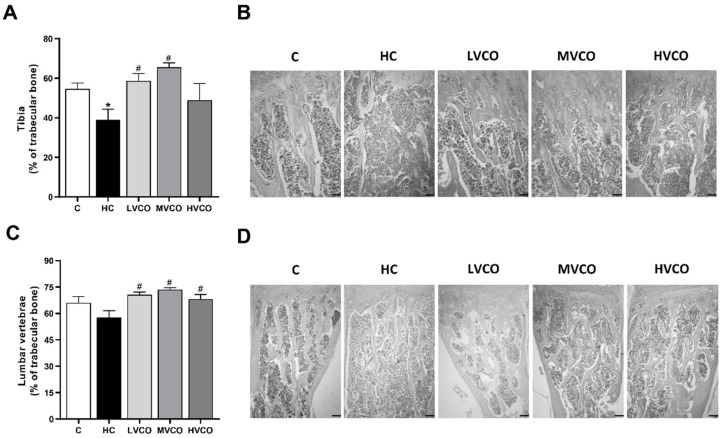
Effect of the HC diet and VCO supplementation on the percentage of trabecular bone in the tibia and lumbar vertebrae. (**A**) The trabecular bone area and (**B**) histological sections of the proximal tibia (scale bars represent 100 µm). (**C**) The trabecular bone area and (**D**) histological sections of lumbar vertebrae (scale bars represent 100 µm). Analyses were performed in mice fed a chow diet, high refined carbohydrate-containing (HC) diet, and HC diet supplemented with 1000 mg/kg (LVCO), 3000 mg/kg (MVCO), or 9000 mg/kg (HVCO) of body weight of virgin coconut oil (VCO). Values are means ± SEM (*n* = 8). * compared with the control (C) group (*p* < 0.05); # compared with HC group (*p* < 0.05).

As VCO supplementation ameliorated the obese phenotype, we evaluated whether this effect also occurred in appendicular and axial bones. HC diet-fed mice showed a lower percentage of trabecular bone in the proximal femur (Figure 1A,B) and the tibia (Figure 2A,B) compared with the control group (C). On the other hand, VCO supplementation at low (LVCO) and medium (MVCO) dosages led to an increase in trabecular bone in these bone sites (Figure 1A,B and Figure 2A,B). All the groups fed with VCO showed a higher percentage of trabecular bone in the distal femur (Figure 1C,D) and lumbar vertebrae (Figure 2C,D) compared with the HC group. 

### 3.2. VCO Supplementation Improves Femur and Alveolar Bone Parameters 

Aiming to better understand how VCO supplementation affects bone integrity, we chose one bone site (metaphyseal region of the femur) to investigate femur quality using micro-CT and mechanical analysis. Although there were no significant differences among groups supplemented with low or medium dosages of VCO, in terms of the percentage of trabecular bone in long bones (tibia and femur) and lumbar vertebrae, we chose only one experimental group (MVCO: 3000 mg/kg BW dosage of VCO) to perform the analysis.

**Figure 3:**
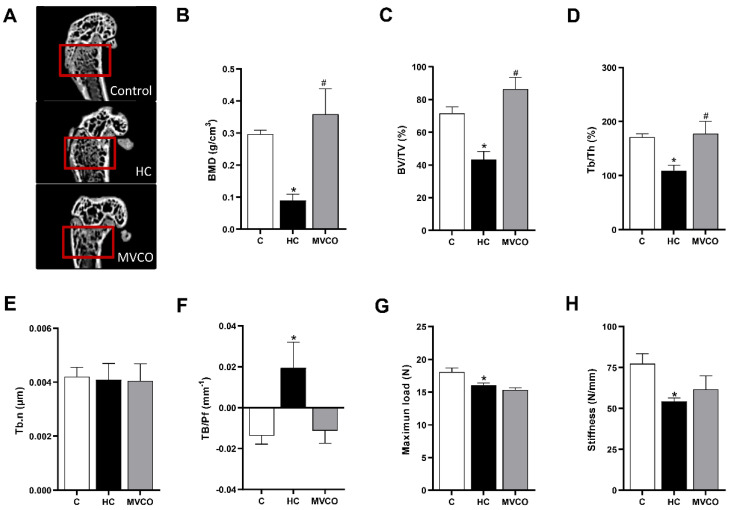
Micro-CT analysis of trabecular bone in the femur. (**A**) Representative femur images (small red squares represent the analyzed region on micro-CT). (**B**) Bone mineral density (BMD), (**C**) trabecular bone volume fraction (BV/TV), (**D**) trabecular thickness (Tb.Th), (**E**) trabecular number (Tb.N), (**F**) trabecular pattern factor (Tb.Pf), (**G**) bone maximum load and (**H**) stiffness of femur in mice fed chow diet (C), high refined carbohydrate-containing diet (HC), and HC diet supplemented with 3000 mg/kg body weight of virgin coconut oil (MVCO). Values are means ± SEM (*n* = 5). * compared with the control (C) group (*p* < 0.05); # compared with HC group (*p* < 0.05).

HC diet-induced osteopenic effects (Figure 3A) on the femur were demonstrated by a decrease in the following bone parameters: BMD (Figure 3B), BV/TV (Figure 3C), Tb.Th (Figure 3D) and an increase in Tb.Pf (Figure 3F). VCO supplementation improved femur microarchitecture compared with the HC group since it leads to a higher BMD (Figure 3A), BV/TV (Figure 3C), and Tb.Th (Figure 3D). There was no statistically significant difference among groups in the Th.N parameter (Figure 3E). A three-point bending flexural test was performed to understand whether the HC diet-induced femur damage impacted mechanical resistance. The maximum load to fracture (Figure 3G) and the femoral stiffness (Figure 3H) were reduced in mice fed with the HC diet. VCO consumption did not prevent the loss of mechanical resistance observed in mice fed the HC diet (Figure 3G,H).

**Figure 4:**
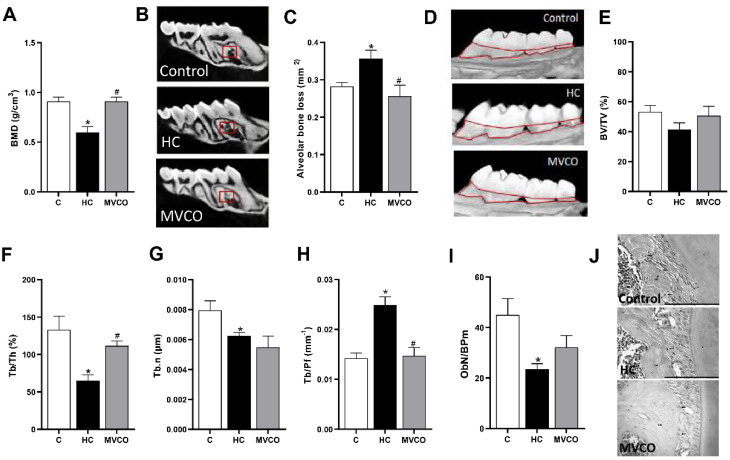
Micro-CT analysis of maxillary bone. (**A**) Bone mineral density (BMD), (**B**) representative images of maxillary (small red squares represent the analyzed region on micro-CT). (**C**) Alveolar bone loss, (**D**) representative images of the maxilla (the area outlined of CEJ-ABC represents the area of alveolar bone resorption). (**E**) Trabecular bone volume fraction (BV/TV), (**F**) trabecular thickness (Tb.Th), (**G**) trabecular number (Tb.N), (**H**) trabecular pattern factor (Tb.Pf) of the maxilla, (**I**) osteoblast number per bone perimeter (ObN/BPm) and (**J**) representative images of osteoblasts in mice fed a chow diet (C), high refined carbohydrate-containing diet (HC), and HC diet supplemented with 3000 mg/kg body weight of virgin coconut oil (MVCO). Values are means ± SEM (*n* = 5). * compared with the control (C) group (*p* < 0.05); # compared with HC group (*p* < 0.05).

The HC diet also negatively affected the alveolar bone in maxillae, as demonstrated by a decrease in BMD (Figure 4A,B), Tb.Th (Figure 4F), and Tb.N (Figure 4G). Furthermore, an increase in horizontal alveolar bone loss (Figure 4C,D) and TB.Pf (Figure 4H) in HC diet-fed mice was also observed. The addition of VCO in the HC diet reversed the osteopenic effects on the alveolar bone by promoting an increase in bone parameters, such as BMD (Figure 4A,B) and Tb.Th (Figure 4F). Additionally, a reduction in horizontal alveolar bone loss (Figure 4C,D) and TB.Pf (Figure 4H) was observed in the MVCO group compared with the HC group. We also evaluated the number of osteoblasts in the alveolar bone. They were reduced in mice fed an HC diet, and no alteration in the VCO supplementation group was observed (Figure 4I,J). 

**Figure 5:**
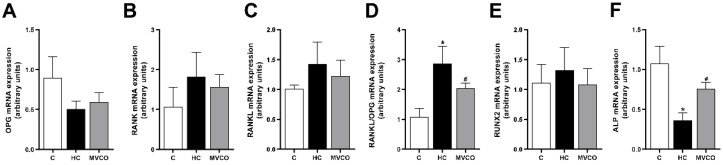
Effect of dietary VCO supplementation in the mRNA expression in the maxilla. (**A**) mRNA expression of Opg, (**B**) receptor activator of nuclear factor κB (Rank) and (**C**) receptor activator of nuclear factor κB ligand (Rankl). (**D**) Rankl/Opg ratio. (**E**) Runx2 and (**F**) Alp. Analyses were performed in mice fed a chow diet (C), high refined carbohydrate-containing diet (HC), and HC diet supplemented with 3000 mg/kg body weight of virgin coconut oil (MVCO). Values are means ± SEM (*n* = 5). * compared with the control (C) group (*p* < 0.05); # compared with HC group (*p* < 0.05).

Moreover, an analysis of the molecules involved in bone remodeling was performed to understand the mechanisms involved in the beneficial bone effects of VCO supplementation. Although no significant differences in the expression of Opg (Figure 5A), Rank (Figure 5B), and Rankl (Figure 5C) were found among groups, a higher Rankl/Opg ratio (Figure 5D) was observed in the HC group when compared with the control group. Interestingly, VCO supplementation was sufficient to decrease this ratio significantly (Figure 5D). Even though the osteoblast markers demonstrated no alteration in Runx2 among the groups (Figure 5E), the Alp expression was reduced in mice fed an HC diet, and VCO supplementation reversed this parameter (Figure 5F).

## 4. Discussion

Specific dietary compounds are associated with beneficial effects on obesity and bone health. VCO intake was associated with body health benefits due to its antioxidant compounds and medium-chain fatty acids (MCFA) [24,25,26,27,28]. In the present study, we evaluated whether VCO intake could help reduce the detrimental bone effects induced by an HC diet intake in a mice model. Herein, our results clearly showed that VCO supplementation promotes (i) amelioration of obese metabolic and inflammatory alterations associated with (ii) a higher percentage of trabecular bone in the tibia, lumbar vertebrae, distal and proximal femur; (iii) improvement in femur microarchitecture, and (iv) higher alveolar bone mass and integrity associated with (v) a lower Rankl/Opg ratio in this bone site and higher Alp expression. 

Traditionally, obesity has been correlated with greater bone mass. It has been postulated that a higher body weight could impose high mechanical stress on the long bones and increase bone mass [40]. However, experimental and clinical studies on obesity [4,5,6,7,8,9,10] have also shown an increase in factors that promote bone catabolism, such as proinflammatory cytokines. Therefore, the negative effect of obesity on bone remodeling appears to outweigh the protective mechanical effect, which is supported by a high incidence of deficient organic matrix mineralization, increased bone fragility, and higher risks of bone fractures in individuals with obesity [5,6]. The higher production of inflammatory cytokines mainly produced by immune cells in obesity could also negatively affect alveolar bone remodeling [10,11,12,13,14,15,16,17]. Therefore, pharmacological and dietary strategies are desired to treat obesity and bone dysfunction.

Our research group previously showed that the HC diet promoted significant osteopenic effects in the femur and alveolar bones [43]. The most plausible explanation for those detrimental bone effects are metabolic and inflammatory disturbances associated with HC diet intake [39,40]. Herein, we confirmed the bone dysfunction induced by the HC diet. The increased leukocyte rolling and levels of proinflammatory cytokines (IL-6 and TNF-α) in adipose tissues, as well as an imbalance of serum concentrations of adipokines (leptin and adiponectin), are outcomes of HC diet consumption [39,40] that could be related with detrimental bone effects. The association between obesity and osteopenic effects is partially explained by a common stem cell precursor leading to the differentiation of adipocytes and osteoblasts. The balance of such differentiation is regulated by several interacting pathways [7,8,9]. Halade et al. (2011) showed that an increased production of inflammatory cytokines (such as IL-1β, IL-6, and TNF-α), as a result of obesity in mice, promotes higher adipogenesis and lower osteoblasts differentiation in the femur [7]. These effects were demonstrated by the up-regulation of Cathepsin k (an osteoclast gene marker) and Rankl expression, and the down-regulation of Runx2/Cbfa1, a transcription factor for osteoblasts differentiation. The detrimental effects of obesity on bone metabolism also affect the alveolar bone. Fujita et al. (2015) showed that obesity triggers mandibular osteoporosis and increases the risk of spontaneous periodontal disease in mice [11]. The enhancement of neutrophil recruitment and oxidative stress, as well as a decrease in antioxidant enzymes in gingival tissue, as a result of adiposity gain, promotes harmful effects on periodontal health [29]. 

When considering bone alterations, our primary purpose was to investigate whether dietary VCO could be an effective strategy to treat or attenuate some bone damage induced by obesity. VCO promoted an increase in trabecular bone in the tibia, lumbar vertebrae, proximal and distal femur and improved the femur microarchitecture. Additionally, VCO intake contributes to long bone maintenance and a greater alveolar bone mass. The maxilla integrity was enhanced by VCO supplementation once it increased BMD and Tb.Th., lowered alveolar bone loss and the Tb.Pf parameter. These effects were associated with a lower Rankl/Opg ratio in the alveolar bone and increased Alp. One way to explain the improvement in bone mass induced by VCO may be its beneficial effect on intermediary metabolism and inflammation. Previous data from our group [43,44] and others [30,32,34] showed that VCO supplementation promoted lower adiposity gain and the amelioration of related metabolic and inflammatory disorders. Indeed, mice fed with VCO showed lower proinflammatory cytokine levels and rolling leukocytes in epididymal adipose tissue. VCO also promotes a reduction in systemic inflammation induced by obesity [44]. In this study, we observed a reduced adipose tissue size associated with a lower leukocyte number caused by VCO treatment. The lower inflammatory response in VCO-fed mice could be due to its effect on adiposity since the expansion of visceral fat tissue leads to the higher recruitment of leukocytes and a wide range of inflammatory mediators [1,2]. The significant presence of phytochemicals (vitamin E, carotenoids, and polyphenols) in VCO [24,25,26,28,29] may have contributed to the decreased lower inflammatory milieu in adipose tissue. These beneficial effects of VCO over obesity may be associated with improved bone health in VCO- fed mice shown herein.

Inflammation induces bone resorption and impairs osteoblastogenesis [7,8,9]. Thus, controlling the inflammatory response may be essential to preventing detrimental bone effects. Accordingly, Sugiura et al. (2012) showed that serum levels of antioxidants and anti-inflammatory carotenoids are inversely associated with lower radial BMD in postmenopausal female subjects [19]. Muhammed et al. (2013) demonstrated that tocotrienol supplementation prevented osteoporotic bone loss in postmenopausal women, and this effect was associated with lower levels of inflammatory cytokines [20]. Improvement of inflammation is also associated with greater alveolar bone integrity [21,22,23]. Supplementation with Omega 3, a fatty acid known for its anti-inflammatory property, reduced alveolar bone loss in rats with periodontitis [21]. Tomofuji et al. (2009) demonstrated that in rats with periodontitis induced by ligature placement, cocoa intake prevented alveolar bone loss by reducing polymorphonuclear leukocyte infiltration and increasing antioxidant defense in periodontal tissues [22,36]. 

Several studies associate VCO consumption with both antioxidant [24,25,26,27,28] and anti-inflammatory [35] effects, which may explain the improvement of bone integrity shown in the present study. Vysakh et al. (2014) demonstrated that the oral supplementation of polyphenolics isolated from VCO in rats with inflammatory arthritis inhibits the expression of inflammatory genes such as COX2, iNOS, TNF-α and IL-6 [35]. In a rat model of osteoporosis, the bone histomorphometry of the femur showed that VCO supplementation prevented bone loss, increased bone volume and trabecular number, and reduced trabecular separation [37]. Abujazia et al. (2012) demonstrated that VCO supplementation prevented lipid peroxidation and increased the antioxidant enzymes in the tibia of osteoporotic rats, and these effects may be correlated with higher bone maintenance and integrity [38]. Additionally, the combined treatment of VCO with tocotrienol-rich fraction in osteoporotic rats appears to be osteoprotective [51].

The major limitation of this study is the intervention duration. An experiment with a more extended intervention period is required to better understand the systemic effect of VCO on healthy bone. Additionally, the doses selected in this study follow previous studies [28,31], and only low and medium dosages promote an increased percentage of trabecular bone in all sites analyzed (tibia, distal and proximal femur and lumbar vertebrae). Long-chain fatty acid (LCFA) may enhance osteoclastogenesis by the up-regulation of Rankl [52]. Therefore, the overload of myristic, palmitic, and stearic (present in approximately 30% of VCO) may have contributed to bone detrimental effects at a high dosage of VCO. 

Animal models represent an essential tool for studying the physiological and molecular events in the development of obesity as they share similar global gene expression patterns with obese humans [1,2,3,6]; therefore, they provide a basic, translational approach in the preclinical setting in elucidating biochemical and physiologic processes. VCO supplementation reverted the bone osteopenic phenotype by increasing the percentage of trabecular bone in multiple bone sites, improving maxillary bone microarchitecture and BMD. However, it is crucial to be cautious about translating animal study results into clinical applications. Although this study elucidated the interesting and consistent beneficial effects of VCO on bone dysfunctions in an animal model, it is necessary to perform clinical studies to confirm these effects on human health. Therefore, clinical studies are required before making recommendations for VCO supplementation.

## 5. Conclusions

Taken together, our data showed that VCO supplementation effectively improved bone structure and prevented bone loss in long bones, lumbar vertebrae, and the maxillary alveolar bone in mice fed with an HC diet. The beneficial effects of VCO on bone microarchitecture may be associated with the promotion of lower adiposity and also the improvement of related metabolic and inflammatory disorders. The significant contents of polyphenols and vitamins in VCO, which exhibit antioxidant and anti-inflammatory properties, may have contributed to greater bone integrity. Thereby, VCO supplementation could be an exciting strategy to prevent bone detrimental effects induced by obesity and its related comorbidities. 

## Figures and Tables

**Table 1:** Analysis of local and systemic changes in control and obese mice treated with virgin coconut oil.

	C	HC	LVCO	MVCO	HVCO
Adiposity index (%)	1.6 ± 0.2	3.1 ± 0.3 *	1.8 ± 0.2 #	1.8 ± 0.1 #	1.7 ± 0.1 #
Serum leptin (ng/mL)	0.4 ± 0.1	2.5 ± 0.5 *	0.8 ± 0.2 #	0.7 ± 0.2 #	0.8 ± 0.3 #
Leukocytes (×10^5^/mL)	90.5 ± 13.7	129.4 ± 23.8 *	62.6 ± 25.4 #	52.2 ± 18.5 #	58.3 ± 21.3 #
Mononuclear (×10^5^/mL)	62.0 ± 16.6	84.9 ± 14.2 *	47.6 ± 18.8 #	49.6 ± 7.4 #	27.7 ± 12.1 #
Neutrophil (×10^5^/mL)	24.5 ± 9.3	44.5 ± 10.5 *	23.7 ± 9.6 #	15.9 ± 5.5 #	30.5 ± 11.1 #

Mice fed a chow diet, high refined carbohydrate-containing (HC) diet, and HC diet supplemented with 1000 mg/kg (LVCO), 3000 mg/kg (MVCO), or 9000 mg/kg (HVCO) of body weight of virgin coconut oil (VCO). Values are means ± SEM (*n* = 8). * compared with the control (C) group (*p* < 0.05); # compared with HC group (*p* < 0.05).

## Data Availability

Data are contained within the article.
